# The Late Dr. Nuttall

**Published:** 1831-01-01

**Authors:** 


### LXXI.

### The Late Dr. Nuttall.

We are glad to see that a subscription has been set on foot for the widow and orphans of the late Dr. Nuttall, by their zealous friend Mr. Tucker. The editor of this Journal having attended Mrs. Nuttall through a long, dangerous, and nearly fatal illness, from which she is now recovering only to mourn over five helpless, but lovely children, can testify conscientiously to the truth of the following passage in Mr. Tuckers's letter, published recently in the Lancet.

"My former letter will partly serve to prove this last assertion. I could add many others of a similar nature, indeed I could fill a small volume with anecdotes of his charitable and other virtuous acts, which I have witnessed and heard of. Scarcely a day passes but I see some poor creature who has benefited by his kindness or professional advice, and who laments with genuine tears his departure from this world. Some may think Dr. Nuttall was indiscriminate in his givings. Not so; no man in London ever witnessed more real misery than he—no man's feelings were ever more tried. He has seen disease, starvation, and every misery, combined in one family; he has visited such at night in the severest weather, all huddled together in a cold desolate room, without a bed to lie upon, a blanket to cover them, or fire to warm their shivering frames. He has felt as every Christian should feel—he has acted as every Christian should act—he has placed fire on their hearths, food in their mouths, and from the dispensary sent medicine to restore the sick member to health. In many cases he has paid for medicines, when delay would have proved dangerous. Who is the man that could condemn him—who is the man that would not applaud him?"

Books of subscriptions are open at the banks of Messrs. Hammersley, Greenwood, and Co. Pall Mall—and also at the Lancet office—Callow and Wilson's—Burgess and Hill—Highley, Fleet-street—and Anderson, Smithfield. We agree with Mr. Tucker, that small sums, on such occasions, would perhaps be better than large, as a far greater number would thus be induced to contribute their mite.

SUBSCRIPTIONS.

Dr. J. Johnson ........ 1*l.* 1*s.* 0*d.*

